# Lung function and airway obstruction: associations with circulating markers of cardiac function and incident heart failure in older men—the British Regional Heart Study

**DOI:** 10.1136/thoraxjnl-2014-206724

**Published:** 2016-01-25

**Authors:** S Goya Wannamethee, A Gerald Shaper, Olia Papacosta, Lucy Lennon, Paul Welsh, Peter H Whincup

**Affiliations:** 1 Department of Primary Care and Population Health, University College London, London, UK; 2 Institute of Cardiovascular & Medical Sciences, BHF Glasgow Cardiovascular Research Centre, University of Glasgow, Glasgow, UK; 3 Division of Population Health Sciences and Education, Population Health Research Centre, St George's University of London, London, UK

**Keywords:** Clinical Epidemiology, COPD epidemiology

## Abstract

**Aims:**

The association between lung function and cardiac markers and heart failure (HF) has been little studied in the general older population. We have examined the association between lung function and airway obstruction with cardiac markers N-terminal pro-brain natriuretic peptide (NT-proBNP) and cardiac troponin T (cTnT) and risk of incident HF in older men.

**Methods and results:**

Prospective study of 3242 men aged 60–79 years without prevalent HF or myocardial infarction followed up for an average period of 13 years, in whom 211 incident HF cases occurred. Incident HF was examined in relation to % predicted FEV1 and FVC. The Global Initiative on Obstructive Lung Diseases spirometry criteria were used to define airway obstruction. Reduced FEV1, but not FVC in the normal range, was significantly associated with increased risk of HF after adjustment for established HF risk factors including inflammation. The adjusted HRs comparing men in the 6–24th percentile with the highest quartile were 1.91 (1.24 to 2.94) and 1.30 (0.86 to 1.96) for FEV1 and FVC, respectively. FEV1 and FVC were inversely associated with NT-proBNP and cTnT, although the association between FEV1 and incident HF remained after adjustment for NT-proBNP and cTnT. Compared with normal subjects (FEV1/FVC ≥0.70 and FVC≥80%), moderate or severe (FEV1/FVC <0.70 and FEV1 <80%) airflow obstruction was independently associated with HF ((adjusted relative risk 1.59 (1.08 to 2.33)). Airflow restriction (FEV1/FVC ≥0.70 and FVC <80%) was not independently associated with HF.

**Conclusions:**

Reduced FEV1 reflecting airflow obstruction is associated with cardiac dysfunction and increased risk of incident HF in older men.

**Key messages**

**What is the key question?**

- What is the association between reduced lung function and mild airflow obstruction with cardiac dysfunction and risk of incident heart failure (HF) in older people.

**What is the bottom line?**

- Reduced lung function reflecting moderate or severe airflow obstruction is associated with increased risk of HF, which is to some extent associated with cardiac dysfunction.

**Why read on?**

- We have shown that the Global Initiative on Obstructive Lung Diseases criteria for defining airflow obstruction based on spirometry can lead to identification of older adults at high risk of HF.

## Introduction

COPD is common in the elderly and is associated with increased cardiovascular (CVD) morbidity and mortality.^1^ ^2^ More recently, it has been established that COPD is common in patients with heart failure (HF) and that patients with COPD are at increased risk of developing HF.^3^ ^4^ The COPD–HF association could reflect shared common risk factors such as smoking, diabetes, obesity and inflammation.^2^ ^3^ ^5^ COPD has also been linked to cardiac stress and myocardial injury.^6–9^ The association between lung function and incident HF, in contrast to CVD,^10^ has been less studied at population level, especially among older people. However, a few prospective population studies have shown an association between reduced lung function as measured by the FEV1, vital capacity (VC) or the FEV1/FVC ratio and incident HF,^11–16^ although this has not been observed in all studies.^17^ Reduced and low normal lung function measured by FEV1 has been associated with increased risk of HF^14–16^ independent of inflammatory markers. The Global Initiative for Chronic Obstructive Lung Diseases (GOLD) classification defines airflow obstruction by FEV/FVC ratio <0.70.^18^ However, normal ageing is associated with lung function declines that often lead to an FEV1/FVC ratio <0.70 for individuals over 65 years, which may lead to misclassification of airway obstruction.^19^ Although one study in the elderly has shown that a low FEV1/FVC ratio (lowest 5th percentile) is associated with increased HF risk,^16^ the risk associated with mild airflow obstruction in the older population is less clear and the relationship between airflow obstruction severity based on the spirometric GOLD classification of lung impairment^18^ and HF has not been studied in the older general population.

High-sensitivity cardiac troponin T (cTnT), a biomarker of cardiomyocyte injury, and N-terminal pro-brain natriuretic peptide (NT-proBNP), a marker of left ventricular wall stress, have both been associated with increased HF risk.^20–22^ NT-proBNP and cTnT have been shown to be raised in patients hospitalised with COPD.^7^ ^8^ ^9^ Whether such changes are seen in those with mild airflow obstruction or low normal lung function has been little studied in the general older population. We, therefore, examined the associations between pulmonary function and risk of HF in older men without prevalent myocardial infarction (MI) or HF and assessed to what extent the association is related to inflammation and cardiac dysfunction (NT-proBNP and cTnT). We have also examined the association between restrictive and obstructive ventilatory patterns with incident HF using a modification of the spirometric GOLD classification for lung function impairment.^17^

## Subjects and methods

The British Regional Heart Study is a prospective study involving 7735 men aged 40–59 years drawn from one general practice in each of 24 British towns, who were screened between 1978 and 1980.^23^ The population studied was socioeconomically representative and comprised predominantly white Europeans (>99%). In 1998–2000, all surviving men, then aged 60–79 years, were invited for a 20th-year follow-up examination, on which the current analyses are based. All men completed a mailed questionnaire providing information on their lifestyle and medical history, had a physical examination and provided a fasting blood sample. The samples were frozen and stored at −20°C on the day of collection and transferred in batches for storage at −70°C until analysis, carried out after no more than one freeze–thaw cycle. The 12-lead ECGs were recorded using a Siemens Sicard 460 instrument and were analysed using Minnesota Coding definitions at the University of Glasgow ECG core laboratory. Men were asked whether a doctor had ever told them that they had angina or MI, HF or stroke; details of their medications were recorded at the examination including use of bronchodilators (British National Formulary code 3.1). In total, 4252 men (77% of available survivors) attended for examination. A total of 3706 men had lung function measures and blood measures of NT-proBNP and cTnT. We excluded men with prior HF or MI (N=464) at examination, leaving 3242 men for analysis.

### CVD risk factor measurements at 1998–2000

Anthropometric measurements including body weight and height were carried out. Details of measurement and classification methods for smoking status, physical activity, social class, alcohol intake, blood pressure and blood lipids in this cohort have been described.^24^ ^25^ C-reactive protein (CRP) (marker of inflammation) was assayed by ultra sensitive nephelometry (Dade Behring, Milton Keynes, UK). NT-proBNP was determined using the Elecsys 2010 (Roche Diagnostics, Burgess Hill, UK).^22^ Troponin T was measured by a high-sensitivity method on an e411 (Roche Diagnostics, Burgess Hill, UK) using the manufacturer’s calibrators and quality control material. The low control CV was 6.6%, the high control CV 3.0% and the assay limit of detection was 3 pg/mL. Electrocardiographic left ventricular hypertrophy (LVH) was defined according to relevant Minnesota codes (codes 3.1 or 3.3). Atrial fibrillation (AF) was defined according to Minnesota codes 8.3.1 and 8.3.3.

### Lung function

FEV1 and FVC were measured using a Vitalograph compact II spirometer with the subject seated. We used predictive equations for FEV1 and FVC derived from the general population Health Survey for England for males aged >25 years to obtain predicted lung function values taking account of age and height.^26^ The following equations were used:

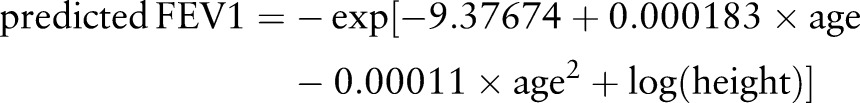


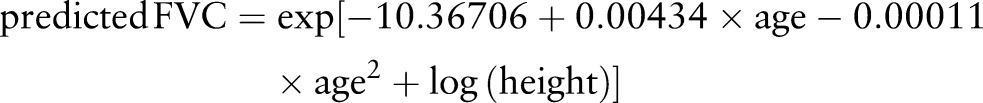


Actual FEV1 and FVC were presented as a percentage of their predicted values. Percent predicted FEV1 (%FEV1)=raw FEV1/predicted FEV1 and per cent predicted FVC (%FVC)=raw FVC/predicted FVC. Abnormal FEV1 (FVC) was defined as men in the lowest 5th percentile of the %FEV (%FVC) distribution. An FEV1/FVC ratio of ≥0.70 was used to define airflow obstruction. We also initially divided the men into five lung pattern groups according to their FEV1/FVC ratio using a modification of the criteria developed by GOLD for defining the severity stages of airflow obstruction^13^: severe airflow obstruction (FEV1/FVC ≥0.70 and FEV1<50% predicted), moderate airflow obstruction (FEV1/FVC<0.70 and FEV1≥50–<80%), mild airflow obstruction (FEV1/FVC<0.70 and FEV1≥80%), restricted (FEV1/FVC≥0.70 and FVC <80% predicted) and normal (FEV1/FVC≥0.70 and FVC≥80% and not on bronchodilators).

### Follow-up

All men were followed up from initial examination (1978–1980) for CVD morbidity^27^; 99% follow-up has been achieved. In the present analyses, all-cause mortality and morbidity events are based on follow-up from re-examination in 1998–2000 to January 2012. Survival times ended at the first HF event or when they were censored for death due to any cause or at the end of the follow-up period (January 2012), whichever occurred first. Information on deaths was collected through the established ‘tagging’ procedures provided by the National Health Service registers. Fatal coronary heart disease (CHD) events were defined as death with CHD (International Classification of Diseases (ICD) 9th revision, codes 410–414) as the underlying code. A non-fatal MI was diagnosed according to WHO criteria. Evidence of non-fatal MI and HF was obtained by ad hoc reports from general practitioners supplemented by biennial reviews of the patients’ practice records (including hospital and clinic correspondence) through to the end of the study period. Incident CHD included fatal CHD and non-fatal MI. Incident non-fatal HF was based on a doctor-confirmed diagnosis of HF from primary care medical records (including hospital and clinical correspondence).^22^ All cases were verified by a review of available clinical information from primary and secondary care records (symptoms, signs, investigations and treatment response) to ensure they are consistent with current recommendations on HF diagnosis.^28^ Incident fatal HF cases were those in which the diagnosis of HF was mentioned as the underlying cause of death at death certificates (ICD 9th revision code 428). Incident HF included both incident non-fatal and fatal HF.

### Statistical methods

The men were initially divided into equal quartiles based on the %FEV1 and %FVC distribution. Because of the interest in those with below normal lung function (lowest 5th percentile), we separated the lowest 5th percentile from the lowest quartile and five groups are used. The study had 80% power to detect a relative risk for HF of 1.6 comparing highest and lowest quartiles of lung function at p<0.05. Tests for trends were carried out fitting FEV1 (FVC) in its original continuous form. Comparisons of baseline characteristics between the FEV1 groups were performed using the χ^2^ test for categorical variables and analysis of variance for continuous variables presented in table 1. The distributions of NT-proBNP, cTnT and CRP were skewed and log transformation was used to normalise these factors. All statistical testing was carried out on log NT-proBNP log cTnT and log CRP. Geometric means were presented for logged variables. Linear regression was used to examine the associations between FEV1 (FVC), log NT-proBNP and log cTnT. Kaplan–Meier methods were used to calculate the cumulative HF incidence for five groups of % predicted FEV1 and % predicted FVC; the log-rank test was used to evaluate differences in the HF rates for these groups. Cox's proportional hazards model was used to assess the HR (relative risk) in a comparison of quarters of %FEV1 and %FVC. The proportional hazards assumption was examined using time-varying covariates, calculating interactions of predictor variables and a function of survival time and including them in the models. Examination of time-varying covariates indicated a violation of the proportionality assumption specifically among the men with abnormal FEV1 or FVC (lowest 5 percentiles) only. We carried out a sensitivity analysis excluding these men as well as truncating follow-up time to 8 years follow-up (the point at which the proportional hazards assumption held for FVC) to assess the robustness of the findings in the men with below normal lung function. We included confounding factors that are known to be associated with HF risk and that were associated with HF in this study in age-adjusted analyses. This included age, body mass index (BMI), social class, smoking, physical activity, heavy drinking, prevalent stroke, diabetes, use of antihypertensive treatment, systolic blood pressure, LVH and AF. In multivariable analyses, smoking (never-smokers, long-term ex-smokers (>15 years), recent ex-smokers (<15 years) and current smokers), social class (manual vs non-manual), physical activity (four groups), diabetes (yes/no), prevalent stroke (yes/no), use of antihypertensive treatment (yes/no), heavy drinking (yes/no), LVH (yes/no) and AF (yes/no) were fitted as categorical variables. Systolic blood pressure, CRP, cTnT and NT-proBNP were fitted as continuous variables. To assess whether the association between lung function and incident HF may be due to the development of incident MI, which in turn results in increased risk of HF, we adjusted for incident CHD by fitting CHD as a time-dependent covariate.

**Table 1:** Baseline characteristics by % predicted FEV_1_ in 3242 men with no prior heart failure or myocardial infarction

	% predicted FEV_1_					
	≤5th percentile	6–24th percentile	25–49th percentile	50–74th percentile	≥75th percentile	p Difference between groups
No. of men	162	651	810	813	806	
Range FEV1, % predicted	<48.4	48.4–75.0	75.1–89.0	89.1–101.6	≥101.7	
FEV (L)	1.12 (0.26)	1.92 (0.31)	2.49 (0.31)	2.86 (0.34)	3.33 (0.47)	
FVC (L)	2.12 (0.09)	2.77 (0.06)	3.27 (0.06)	3.64 (0.06)	4.13 (0.07)	
% FEV1/FVC<0.7	33.3	34.1	30.3	26.8	21.1	
Categorical variables (%)						
Smokers	27.2	21.4	13.8	8.5	4.7	<0.001
Manual	63.6	62.5	54.0	51.3	45.8	<0.001
Inactive	54.4	39.0	31.6	30.8	26.2	<0.001
Heavy drinkers	6.2	5.2	3.2	4.2	3.0	0.083
Diabetes	14.8	16.0	12.4	10.8	10.4	0.001
Stroke	6.8	5.5	5.1	2.8	3.4	0.017
AF	3.7	3.5	3.5	2.8	2.4	0.620
Antihypertensive use	33.3	34.1	30.3	26.8	21.1	<0.001
Left ventricular hypertrophy	5.6	6.3	7.3	8.9	8.2	0.302
Continuous variables (means)						
Age (years)	69.4 (5.4)	69.1 (5.4)	68.0 (5.2)	67.8 (5.5)	68.9 (5.7)	<0.001
BMI (kg/m^2^)	25.5 (3.9)	27.1 (4.0)	27.1 (3.7)	26.9 (3.3)	26.4 (3.2)	<0.001
Systolic blood pressure (mm Hg)	147.8 (21.9)	151.5 (25.4)	149.2 (23.5)	150.8 (24.1)	148.7 (22.9)	0.090
Cholesterol (mmol/L)	5.86 (1.10)	5.93 (1.02)	6.10 (1.10)	6.09 (1.04)	6.09 (1.06)	0.002
High-density lipoprotein-cholesterol (mmol/L)	1.41 (0.41)	1.31 (0.35)	1.32 (0.34)	1.31 (0.33)	1.36 (0.33)	0.002
C-reactive protein (mg/L)*	4.48 (1.30–6.01)	2.27 (1.08–4.82)	1.82 (0.90–3.47)	1.38 (0.73–2.87)	1.23 (0.60–2.40)	<0.001
N-terminal pro-brain natriuretic peptide (pg/mL)*	115.6 (55–257)	108.9 (49–220)	86.5 (44–160)	77.5 (38–147)	80.6 (41–146)	<0.001
Cardiac troponin T (pg/mL)*	13.7 (10.1–18.9)	12.4 (9.3–16.4)	11.2 (8.6–15.9)	11.0 (8.5–14.8)	11.0 (8.2–14.7)	<0.001

Mean and SD unless specified.

*Geometric mean and IQR.

## Results

### Baseline characteristics by FEV1

Table 1 shows baseline characteristics in the study population by the five FEV_1_ groups. Poorer lung function was associated with several adverse CV risk factors including smoking, physical inactivity, diabetes, inflammation (CRP) and cardiac dysfunction (NT-proBNP, cTnT).

### Lung function and cardiac markers

Table 2 shows the associations between %FEV_1_, %FVC and cardiac markers adjusted for potential confounders and inflammation. %FEV_1_ and, to a lesser extent, %FVC were significantly associated with NT-proBNP. Strong inverse associations were seen between both %FEV_1_ and %FVC with cTnT after adjustment. However, when both were included in the model FEV1 related only to NT-proBNP, whereas FVC related only to cTnT.

**Table 2:** Associations of FEV1 and FVC (expressed as % of predicted values) with log-transformed N-terminal pro-brain natriuretic peptide (NT-proBNP) and cardiac troponin T (cTnT) in men with no prior heart failure or myocardial infarction

	FEV_1_	p Value	FVC β coefficients (95% CI)	p Value
Log NT-proBNP (pg/mL)	Change in log NT-pro BNP per increase in 10 percentile change in FEV1		Change in log NT-pro BNP per increase in 10 percentile change in FVC	
Age-adjusted	−0.054 (−0.070 to −0.038)	<0.001	−0.053 (−0.071 to −0.035)	<0.001
Model 1	−0.034 (−0.050 to −0.018)	<0.001	−0.029 (−0.048 to −0.011)	<0.001
Model 2	−0.023 (−0.039 to −0.007)	0.005	−0.021 (−0.037 to −0.003)	0.026
Model 3	−0.024 (−0.048 to 0.000)	0.050	0.00008 (−0.028 to 0.029)	0.950
Log cTnT (pg/mL)	Change in log cTnT increase in 10 percentile change in FEV1		Change in log CTnT per increase in 10 percentile change in FVC	
Age−adjusted	−0.024 (−0.032 to −0.016)	<0.001	−0.033 (−0.041 to −0.025)	<0.001
Model 1	−0.024 (−0.032 to −0.016)	<0.001	−0.030 (−0.038 to −0.022)	<0.001
Model 2	−0.021 (−0.029 to −0.013)	<0.001	−0.027 (−0.035 to −0.019)	<0.001
Model 3	−0.004 (−0.014 to 0.006)	0.450	−0.024 (−0.036 to −0.012)	0.002

Model 1=adjusted for age, smoking status, physical activity, heavy drinking, social class, body mass index, left ventricular hypertrophy, systolic blood pressure, use of antihypertensive drugs, diabetes, stroke and atrial fibrillation.

Model 2=model 1+ C-reactive protein (CRP).

Model 3=Model 1+CRP+FEV and FVC.

### Lung function and incident HF

**Figure 1:**
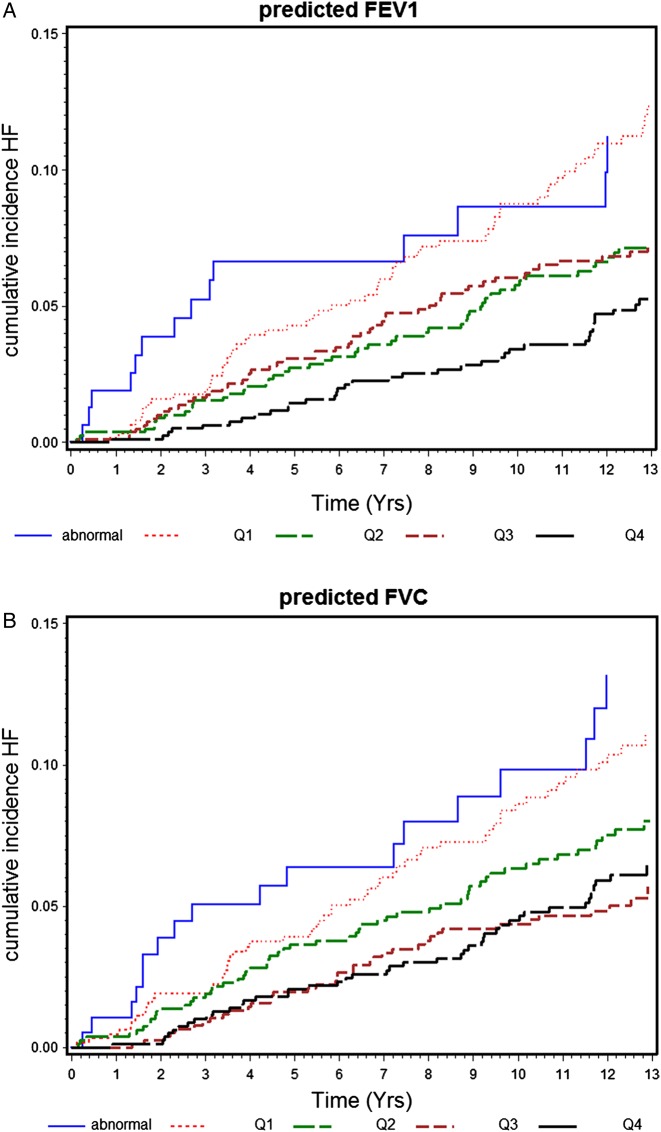
(A and B) Cumulative heart failure (HF) incidence by five groups of % predicted FEV1 and % predicted FVC in 3242 men without prevalent myocardial infarction or HF.

During the mean follow-up period of 13 years, there were 211 incident HF cases (rate 6.1/1000 person-years) in the 3242 men with no prior HF or MI at baseline. Figure 1 shows the cumulative incidence of HF by the five groups of %FEV_1_ and %FVC in these men. Risk of HF increased with decreasing %FEV1 and %FVC. Table 3 shows the association between lung function and incident HF, with adjustment for potential confounders and mediators. The increased risk associated with low lung function remained even after adjustment for age, smoking, physical activity, social class, alcohol intake, BMI, antihypertensive treatment, systolic blood pressure, prevalent diabetes, stroke, AF, LVH and CRP. Adjustment for NT-proBNP and cTnT attenuated the increased HF risk, but it remained significant in those in the lowest quartile within the normal range. Weaker associations were seen with FVC, with HF risk only elevated in those substantially below the normal range, but this was attenuated after adjustment for cardiac markers.

**Table 3:** Adjusted HR (95% CIs) for incident heart failure (HF) in relation to % predicted FEV1 and % predicted FVC in men with no prior HF or myocardial infarction

	Abnormal (≤5th percentile)	6–24th percentile	25–49th percentile	50–74th percentile	≥75th percentile	p Trend
% predicted FEV						
No. of men	162	651	810	813	806	
Rate/1000 (n)	10.1 (14)	9.4 (60)	5.7 (49)	5.9 (52)	4.0 (36)	
Age-adjusted	2.76 (1.49 to 5.12)	2.44 (1.61 to 3.69)	1.59 (1.03 to 2.45)	1.64 (1.07 to 2.51)	1.00	<0.001
Model 1	2.19 (1.14 to 4.21)	1.96 (1.27 to 3.02)	1.34 (0.89 to 2.08)	1.45 (0.94 to 2.23)	1.00	0.002
Model 2	2.10 (1.10 to 4.05)	1.91 (1.24 to 2.94)	1.31 (0.84 to 2.03)	1.43 (0.93 to 2.19)	1.00	0.004
Model 3	1.87 (0.97 to 3.60)	1.70 (1.10 to 2.63)	1.25 (0.80 to 1.93)	1.36 (0.89 to 2.10)	1.00	0.016
Model 4	1.78 (0.93 to 3.44)	1.72 (1.11 to 2.65)	1.18 (0.76 to 1.84)	1.41 (0.92 to 2.13)	1.00	0.024
Model 5	1.63 (0.84 to 3.19)	1.61 (1.04 to 2.49)	1.17 (0.75 to 1.82)	1.35 (0.88 to 2.07)	1.00	0.047
% predicted FVC						
No. of men	164	649	813	807	811	
Rate/1000 (n)	11.3 (16)	8.8 (56)	6.4 (56)	4.5 (38)	4.9 (45)	
Age-adjusted	2.29 (1.37 to 4.18)	1.80 (1.22 to 2.67)	1.38 (0.93 to 2.04)	0.91 (0.59 to 1.40)	1.00	<0.001
Model 1	1.78 (0.98 to 3.22)	1.33 (0.88 to 2.00)	1.14 (0.77 to 1.70)	0.80 (0.52 to 1.23)	1.00	0.083
Model 2	1.68 (0.92 to 3.05)	1.30 (0.86 to 1.96)	1.13 (0.76 to 1.68)	0.79 (0.31 to 1.22)	1.00	0.121
Model 3	1.50 (0.82 to 2.75)	1.33 (0.88 to 2.00)	1.11 (0.74 to 1.65)	0.84 (0.55 to 1.31)	1.00	0.213
Model 4	1.46 (0.80 to 2.67)	1.18 (0.79 to 1.78)	1.07 (0.71 to 1.59)	0.77 (0.50 to 1.20)	1.00	0.386
Model 5	1.29 (0.69 to 2.39)	1.25 (0.83 to 1.88)	1.07 (0.72 to 1.60)	0.83 (0.53 to 1.20)	1.00	0.453

Model 1=adjusted for age, smoking status, physical activity, heavy drinking, social class, body mass index, left ventricular hypertrophy, systolic blood pressure, use of antihypertensive drugs, diabetes, stroke and atrial fibrillation.

Model 2=model 1+ C-reactive protein.

Model 3=model 2+ N-terminal pro-brain natriuretic peptide (NT-proBNP ).Model 4=model 2+ cardiac troponin T (cTnT).

Model 5=model 2+NT-proBNP+cTnT.

The same pattern of association was seen even when analyses were restricted to never-smokers and long-term ex-smokers (>20 years since quitting) (N=2222; 138 cases). Men with low normal FEV showed significantly increased risk after adjustment for confounders and CRP (model 2) (HR 2.00 (1.20 to 3.34)). The increased risk remained after adjustment for NT-proBNP and cTnT, though it was attenuated (HR 1.66, 95% CI (0.99 to 2.78)). No association was seen with men with low normal FVC (HR 1.43, 95% CI (0.86 to 2.38)) after adjustment for confounders and CRP (model 2).

Exclusion of men with abnormal FEV1 reduced the higher risk in those with low FVC even further. We also carried out sensitivity analysis truncating follow-up time to 8 years because of the violation of the proportional hazards in those with abnormal lung function with 13 years follow-up. Although the numbers were smaller (N=137 cases), the pattern of relation remained the same in that FVC showed no independent association after full adjustment (model 5) and the increased risk in those with abnormal FVC was markedly attenuated after adjustment for cardiac markers. The HR for those with abnormal FVC adjusted for the factors in model 1 was 2.88 (0.94 to 4.44), and this was attenuated to 1.33 (0.60 to 2.93) after full adjustment (model 5). For abnormal FEV1, the corresponding HR was 2.77 (1.26 to 6.09) and 1.99 (0.89 to 4.43), respectively. Men with low FEV1 in the 6th–24th percentile still showed significantly increased risk after full adjustment (model 5) (HR, 1.77 95% CI (1.00 to 3.13)).

### Restrictive and obstructive lung function patterns

#### Cardiac function

Compared with the normal group, NT-proBNP was raised in men with moderate or severe airway obstruction but not in those with mild airway obstruction; CTnT was only raised in those with severe airway obstruction (table 4). Both NT-proBNP and CTnT were raised in men with restrictive patterns even after adjustment for confounders.

**Table 4:** Mean lung function and the difference in mean level of log N terminal pro-brain natriuretic peptide (NT-proBNP) and log cardiac troponin T (cTnT) in men with restrictive and obstructive lung patterns compared with men with normal lung pattern in men with no prior heart failure or myocardial infarction

			Obstructive		
	Normal	Restrictive	Mild	Moderate	Severe
	N=1582	N=887	N=235	N=351	N=137
Mean FEV1,% predicted	101	75	95	66	39
Range, %	74–193	15–106	88–219	55–122	29–125
Mean FEV1(SD), L	3.04 (0.48)	2.25 (0.49)	2.80 (0.53)	1.96 (0.34)	1.14 (0.27)
Mean FVC, % Predicted	96	67	101	83	61
Range, %	80–156	12–79	88–219	55–122	29–125
Mean FVC (SD), L	3.82 (0.59)	2.67 (0.56)	4.32 (0.80)	3.25 (0.61)	2.39 (0.83)
		Mean difference (95% CI) in log NT-proBNP (pg/mL)			
Age-adjusted	0	0.16 (0.08 to 0.24)*	−0.03 (−0.17 to 0.11)	0.20 (0.19 to 0.11)*	0.21 (0.04 to 0.39)*
Model 1	0	0.12 (0.04 to 0.20)*	−0.05 (−0.17 to 0.08)	0.16 (0.05 to 0.27)*	0.17 (0.01 to 0.32)*
Model 2	0	0.08 (0.003 to 0.16)*	−0.06 (−0.19 to 0.07)	0.11 (0.002 to 0.22)*	0.08 (−0.09 to 0.25)
		Mean difference (95% CI) in log cTnT (pg/mL))			
Age-adjusted	0	0.10 (0.07 to 0.14)*	−0.02 (−0.08 to 0.37)	0.00 (−0.05 to 0.05)	0.11 (0.04 to 0.19)*
Model 1	0	0.08 (0.06 to 0.10)*	−0.02 (−0.08 to 0.04)	−0.01 (−0.05 to 0.04)	0.11 (0.04 to 0.19)*
Model 2	0	0.07 (0.05 to 0.09)*	−0.02 (−0.08 to 0.04)	−0.01 (−0.06 to 0.04)	0.08 (0.004 to 0.16)*

Normal used as the reference group.

Model 1=adjusted for age, smoking status, physical activity, heavy drinking, social class, body mass index, left ventricular hypertrophy, systolic blood pressure, use of antihypertensive drugs, diabetes, stroke and atrial fibrillation.

Model 2=model 1+ C-reactive protein.

*Significantly different from the normal group (p<0.05); normal=FEV1/FVC≥0.70 and FVC≥80% and not on bronchodilators.

#### Incident HF

As the number of men with severe airflow obstruction was small and they showed similar risk to those with moderate airflow obstruction, we combined men with moderate and severe airflow obstruction and four groups are used (table 5). Restrictive lung impairment was associated with increased risk of HF, which was markedly attenuated after adjustment for HF risk factors and inflammation. HF risk was significantly increased in those with moderate or severe airflow obstruction (but not mild airflow obstruction) even after adjustment for established HF risk factors, CRP and cardiac markers (NT-proBNP and cTnT). Further adjustment for incident CHD made little difference. The increased risk seen was apparent even when those with severe obstruction were excluded (adjusted HR (model 5) 1.56 (1.03 to 2.37)).

**Table 5:** Adjusted HR (95% CIs) for incident heart failure (HF) in relation to restrictive and obstructive lung patterns in men with no prior HF or myocardial infarction

	Normal N=1582	Restrictive N=887	Mild N=235	Moderate/severe# N=488
Incident HF				
Rate/1000 (n)	4.6 (81)	7.5 (67)	5.1 (13)	9.2 (43)
Age-adjusted	1.00	1.59 (1.15 to 2.19)	0.93 (0.52 to 1.68)	1.86 (1.28 to 2.69)
Model 1	1.00	1.35 (0.97 to 1.88)	1.00 (0.56 to 1.82)	1.70 (1.16 to 2.50)
Model 2	1.00	1.32 (0.94 to 1.84)	0.98 (0.54 to 1.77)	1.67 (1.14 to 2.46)
Model 3	1.00	1.24 (0.89 to 1.73)	0.92 (0.51 to 1.68)	1.58 (1.07 to 2.33)
Model 4	1.00	1.19 (0.85 to 1.68)	0.99 (0.55 to 1.79)	1.66 (1.13 to 2.45)
Model 5	1.00	1.15 (0.82 to 1.62)	0.93 (0.51 to 1.68)	1.59 (1.08 to 2.33)

Model 1=adjusted for age, smoking status, physical activity, heavy drinking, social class, body mass index, left ventricular hypertrophy, systolic blood pressure, use of antihypertensive drugs, diabetes, stroke and atrial fibrillation.

Model 2=model 1+ C-reactive protein.

Model 3=model 2+ N-terminal pro-brain natriuretic peptide (NT-proBNP).

Model 4=model 2+ cardiac troponin T (cTnT).

Model 5=model 2+NT-proBNP+cTnT.

*Moderate and severe group were combined due to small numbers.

## Discussion

In this study of older British men with no prior HF or MI, reduced FEV1 even within the low normal range was associated with increased HF risk after adjustment for a wide range of established HF risk factors and inflammation. Reduced FVC within the normal range, however, was not associated with increased risk of HF after adjustment; risk was only increased in those with exceptionally low FVC (lowest 5th percentile). Our findings confirm previous studies in middle-aged and older populations showing an inverse association between FEV1 and incident HF.^14–16^ The findings were more evident for airflow obstruction airways rather than restrictive lung impairment. Our findings extend previous studies by examining the association with cardiac markers NT-proBNP and cTnT not previously assessed and examining the spirometric GOLD criteria for obstructive airways in relation to HF risk in an elderly population.

### Lung function and incident HF

The increased HF risk associated with reduced FEV1 in this study was not explained by established HF risk factors known to be associated with lung function, including LVH and AF,^29–31^ which is consistent with findings from the Cardiovascular Health Study.^12^ The association between FEV1 and HF was independent of inflammatory markers CRP and interleukin-6 and adjustment for incident CHD did not alter the findings as shown in other studies.^12^ ^14^ ^15^ Shortness of breath and low lung function is a symptom of both COPD and HF. There is thus the possibility that poor lung function is reflecting preclinical HF. However, we were able to adjust for cardiac markers of HF including NT-proBNP and cTnT and the association between low FEV1 and HF persisted after these adjustments. Although reduced FVC (reflecting restrictive patterns of lung disease) was associated with HF, this was markedly attenuated after adjustment for established HF risk factors. The lack of association between FVC and HF is unlikely to be the result of competing non-cardiac causes as FVC and FEV1 were both related to non-cardiac causes and censoring for non-cardiac causes had only minimal effect.

### Restrictive and obstructive patterns and incident HF

Although a few population studies have shown associations between both restrictive and obstructive lung function patterns and HF after adjustment for established HF risk factors,^14^ ^15^ we have observed a weaker association between restrictive lung function patterns and risk of HF than with obstructive patterns, consistent with the weaker findings seen for FVC than the FEV1. Restrictive lung function patterns (FEV1/FVC>0.70 and predicted FVC<80%) were common in this elderly study population (27%) but were not associated with increased risk of HF after adjustment for established HF risk factors. Normal ageing is strongly associated with lower FVC and reduced FVC may not actively identify true pulmonary restriction in the elderly.^32^ Although low FVC may identify restrictive lung patterns in the elderly in the presence of a normal FEV1/FVC ratio (>0.7), few men had normal FEV1/FVC and a low FVC in this study and we did not have the power to detect a significant difference in this group. In contrast, moderate or severe airway obstruction (FEV1/FVC ratio <0.7 and FEV1<80%) was significantly associated with increased HF risk, an increase that was not explained by established risk factors or inflammation**.** Mild airway obstruction was not associated with increased risk; this may relate to the fact that mild obstruction had no effect on cardiac function as was seen in men with moderate/severe airway obstruction.

### Reduced lung function and cardiac dysfunction

We have shown strong inverse associations between both FEV1 and FVC and cTnT and NT-proBNP. Increased levels of cTnT were more strongly associated with restrictive lung function patterns and were only markedly raised in the small group of men with very severe airways obstruction, consistent with the findings of studies in hospitalised patients with COPD.^6–9^ NT-proBNP was, however, related to both obstructive and restrictive airway patterns. NT-proBNP is released from cardiac myocytes in response to increased ventricular wall stress and cardiac dysfunction.^33^ Several mechanisms could explain the increased NT-proBNP in subjects with COPD. Airway obstruction decreases expiratory flow rates and causes lung hyperinflation, which is associated with decreased cardiac function and may increase NT-proBNP.^6^ NT-proBNP may also be elevated due to pulmonary hypertension and right ventricular dysfunction caused by pulmonary arterial pressure overload.^34^ ^35^ Early right ventricular structural abnormalities could ultimately lead to a deterioration of left ventricular function and clinical HF.

The association between reduced FEV1 airway obstruction and incident HF was partially attenuated by adjustment for baseline NT-proBNP and cTnT, although risk still remained significantly increased. Although NT-proBNP and cTnT contribute to the association between airflow obstruction and HF risk, other mechanisms including oxidative stress have also been implicated.^16^

## Strengths and limitations

The strength of the study is as a representative cohort with good standardised lung function measures with a wide range of HF risk factors measured. However, it was based on an older, predominantly white male population of European extraction, so that the results cannot be generalised directly to women, younger populations or other ethnic groups. Like most epidemiological studies, we did not have post-bronchodilator lung function, which is used for the strict classification using GOLD criteria. Nevertheless, using the criteria for moderate or severe airway obstruction based on pre- bronchodilator lung function, we have shown airway obstruction to be associated with increased HF risk. The current findings are based on doctor-diagnosed HF, which may underestimate the true incidence of HF in this study population. However, the determinants of HF in this study population (including obesity, NT-proBNP, social class and heavy drinking)^22^ ^24^ ^36^ ^37^ generally accord with prior data and suggest that the HF outcome used was valid. Adjustments were based on measurements at examination, and we had no information on incident AF, which is associated with reduced lung function and HF risk. Information on echocardiogram measurements was not available in all men, and we were not, therefore, able to differentiate systolic and diastolic HF.

## Conclusion

Reduced FEV1, reflecting moderate or severe airflow obstruction, is associated with elevated levels of cardiac dysfunction and increased risk of HF in older men. The use of GOLD criteria for defining airflow obstruction based on spirometry can lead to identification of older adults at high risk of HF. Spirometry measurements might be considered in clinical practice to identify older people at high risk of developing HF who may benefit from further cardiac investigations and potentially benefit from early interventions.
